# Effects of dietary supplementation with butyrate glycerides on lipid metabolism, intestinal morphology, and microbiota population in laying hens^✰^

**DOI:** 10.1016/j.psj.2024.104755

**Published:** 2025-01-24

**Authors:** Minyao Zhou, Yanqiu Luo, Ji Qiu, Haidong Wang, Xinyu Li, Kexin Zhang, Xiaoteng Li, Muhammad Umar Yaqoob, Minqi Wang

**Affiliations:** College of Animal Science, Zhejiang University, Hangzhou 310058, China

**Keywords:** Butyrate glycerides, Lipid metabolism, Intestinal morphology, gut microbiota, Laying hens

## Abstract

The present study investigated the impact of butyrate glycerides (**BG**) on lipid metabolism, intestinal morphology, and microbiota of laying hens. Four hundred eighty 54-week-old Hy-line Brown laying hens were randomly selected and divided into five groups. The control group (**ND**) was fed a basal diet. Meanwhile, the remaining groups were given a basal supplemented with 0.5, 1, 2, and 4 g/kg of the product containing BG and were designated as **BG-0.5, BG-1, BG-2**, and **BG-4** groups, respectively. The findings showed that: (1) BG supplementation significantly decreased (*P* < 0.001) the blood Glu levels (BG-0.5, BG-1, BG-2, and BG-4) and increased (*P* < 0.001) the serum HDL-C levels (BG-2, and BG-4). (2) The BG-2 and BG-4 groups showed an increase (*P* < 0.01) in abdominal lipid HSL activity. (3) The levels of hepatic TC and TG in all BG groups were significantly decreased (*P* < 0.05). (4) The addition of BG resulted in a significant reduction in the mRNA expression of the liver X receptor alpha (LXRα) (*P* < 0.05). (5) All BG groups presented a substantial reduction in duodenal crypt depth and a notable increase in the ratio of villus height to crypt depth (V/C) (*P* < 0.01). Additionally, all BG groups exhibited a significant increase in villus height in the ileum (*P* < 0.001). (6) Both the BG-1 and BG-4 groups exhibited a significant reduction in the amounts of n-butyric and n-glutaric acids in the cecum contents (*P* < 0.05). (7) The inclusion of BG did not substantially impact the diversity of cecal microbiota in laying hens. However, it dramatically boosted the proportion of the beneficial bacterium *Alistipes* (*P* < 0.05) and reduced the abundance of the harmful bacterium Verrucomicrobiota (*P* < 0.05). Overall, incorporating BG with glycerol monobutyrate as the diet's primary active component reduces fat accumulation in laying hens' blood and liver. It potentially regulates lipid metabolism via the PPARγ-LXRα-SREBP1c pathway. Additionally, BG has the potential to enhance the structure of the small intestine's mucous membrane and increase the presence of beneficial bacteria. Under the experimental conditions, late-laying hens supplemented with 4 g/kg BG performed best overall.

## INTRODUCTION

In the modern layer industry, hens are reared in conventional cage facilities. However, confining hens in cages can lead to fat metabolism disorders such as fatty liver hemorrhagic syndrome (**FLHS**), which is more prevalent in laying hens during the later stages of their laying cycle (Shini et al., 2019). FLHS can significantly impact hens' laying performance and egg quality (Chen et al., 2022; Tao et al., 2022). Studies have indicated that over 40 % of deceased hens in commercially caged hens die due to FLHS (Shini et al., 2019), resulting in substantial financial losses in modern intensive poultry farming. Therefore, it is imperative to effectively regulate lipid metabolism to minimize the incidence of FLHS in caged laying hens by reducing fat accumulation in the liver. This is crucial for improving the performance and overall health of the hens (Han et al., 2023).

Glycerol monobutyrate (also known as 1-Butyryl-Glycerol, Mono butyrin, MB) was initially discovered while isolating differentiation-dependent angiogenic factors released by adipocytes (Dobson et al., 1990). Glycerol monobutyrate has been proven to be an effective anticoccidial agent in broilers (Antongiovanni et al., 2016). It significantly inhibits *Clostridium perfringens* and *Salmonella typhimurium* in poultry (Namkung et al., 2011). Additionally, it enhances the intestinal barrier function in swine (Kovanda et al., 2020). Glycerol monobutyrate also has a notable impact on muscle development and lipid deposition in broiler chickens (Bedford et al., 2017), and it reduces abdominal fat deposition in broilers in a dose-response manner (Bedford et al., 2018). Feng et al. (2021) found that supplementing the ration with 250 mg/kg of glycerol monobutyrate improved egg weight and potentially decreased egg breakage during the late laying period. Nguyen et al. (2017) proposed that glycerol monobutyrate effectively mitigated lipid metabolic problems and decreased intestinal barrier function caused by high-fat diets. This was accomplished by reducing hepatic fat and succinic acid levels and improving the intestinal microbiota in high-fat rats (Nguyen et al., 2017; Nguyen et al., 2019; Nguyen et al., 2020).

Nonetheless, using pure glycerol mono butyrate on an industrial scale is very expensive. So, it is better to use a manufacturer's glycerol product to figure out the best amount of glycerol mono butyrate to use. BalanGut LS P (**BG**) (BASF, Germany) is a feeding additive complex that includes glycerol mono butyrate, glycerol dibutyrate, and glycerol caprylate as its primary active ingredients.

This experiment aimed to investigate the impact of incorporating varying levels of BG into the diet of laying hens during the late laying phase, especially in analyzing the effects on lipid metabolism, intestinal mucosa morphology, and intestinal flora. The objective was to determine the optimal level of BG to be added to the diet of laying hens and to provide a scientific basis for its widespread use in their diet. Additionally, the findings may contribute to developing interventions for fatty liver in humans.

## MATERIALS AND METHODS

### Birds, Diets, and Experimental Procedures

The experiment was conducted in the Yangcunqiao Laying Hens Breeding Community in Jiande, Hangzhou City, Zhejiang Province, China. The Animal Experimentation Centre of Zhejiang University approved the experimental protocols utilized in the study 29081. A total of 480 Hy-lind Brown laying hens, aged 54 weeks were randomly assigned to five treatment groups. Each treatment group consisted of six replicates, with 16 birds in each replication (four cages, with four chickens per cage). T1: control group (**ND**), fed a basal diet; T2: **BG-0.5** group; T3: **BG-1** group; T4: **BG-2** group; T5: **BG-4** group; fed basal diet supplemented with 0.5, 1, 2, and 4 g/kg of BalanGut LS P, respectively. BalanGut LS P was provided by BASF Germany (Table 1). The basal diet was a corn-soybean meal-based diet formulated as per recommendations of NRC (1994) and Hy-Line W-36 Management Guide (Hy-Line International, 2007), with necessary modifications to suit local production conditions. Table 2 shows the composition and nutrient levels of the diet. The hens were given an adaptation period of 1 week, during which all hens were fed the basal diet. At the same time, adjustments were made to the experimental hens to ensure no significant differences in body weight and egg production rate between replicates at the start of the regular trial period of 5 weeks.

**Table 1:** Composition and specification of BalanGut LS P.

Composition	Proportions
Monoglycerides, diglycerides, and triglycerides of butyric, caprylic, and capric acids	43 to 49 %
Glycerol	16 to 22 %
SiO_2_	31 to 34 %
Free fatty acid	< 1 %
Specification	
Total glycerides of butyric acid and medium-chain fatty acids	43 to 49 %
Butyric acid content	17 to 21 %
Medium chain fatty acids (C8^1^, C10^2^) content	5.0 to 8.0 %

^1^ Medium chain fatty acids of eight carbon atoms.

^2^ Medium chain fatty acids of ten carbon atoms.

**Table 2:** Ingredient and nutrient composition of basal diet.

Ingredients	Proportions (%)	Nutrients (%)	Levels
Corn	63.00	Metabolic energy 2(MJ/kg)	11.52
Soybean meal	25.00	Crude protein	16.25
Oystershellmeal	8.00	Lysine	0.80
Limestone	1.80	Methionine	0.41
Calcium biphosphate	1.20	Ca	4.10
Salt	0.20	Total P	0.63
Sodium bicarbonate	0.30		
Methionine	0.10		
Choline (50 %)	0.10		
Premix 1	0.30		
Total	100 %		

1 The premix provides each kg of ration vitamin A 8000 IU, vitamin D 1600 IU, vitamin E 5 mg, vitamin K 0.5 mg, thiamin 0.8 mg, riboflavin 2.5 mg, pantothenic acid 2.2 mg, niacin 20 mg, pyridoxine 3 mg, biotin 0.1 mg, folic acid 0.25 mg, vitamin B_12_ 0.01 mg, Cu 10 mg, Fe 90 mg, Mn 125 mg, Zn 70 mg, I 0.5 mg, Se 0.15 mg.

2 Metabolic energy is calculated, and other nutritional values are analyzed data.

The experiment was carried out using a configuration of three levels of metal cages that were piled on top of each other. On one side, there were four cages per level, each containing 4 hens. The stocking density was 12 birds/m^2^. During the feeding trial, hens were manually supplied with feed twice daily (7:00 a.m. and 2:00 p.m.). Additionally, a nipple drinking system was installed with cages to provide clean and fresh water around the clock. Eggs were collected daily at 3:00 p.m. A lighting schedule of 16 h of light and 8 h of darkness was followed, and the temperature in the hen house was maintained at 25 ± 3 °C.

### Blood Serum Biochemical Analysis

On the 35^th^ day, two birds were selected from each replicate, resulting in a total of 12 chickens per group and a total of 60 chickens. Following 24 h of fasting, blood was obtained from the jugular vein and transferred to a centrifuge tube designed for coagulation. The tube was centrifuged (3000 rpm for 10 min at 4 °C). After the serum had settled at room temperature, it was carefully transferred into Eppendorf tubes. Blood serum was analyzed for concentrations or activity levels of glucose (**Glu**), total cholesterol (**TC**), triglyceride (**TG**), lipase (**Lip**), free fatty acid (**NEFA**), and alkaline phosphatase (**ALP**) using an automatic biochemical analyzer (Olympus AU2700, Japan). High-density lipoprotein cholesterol (**HDL-C**) and low-density lipoprotein cholesterol (**LDL-C**) were measured using test kits provided by Nanjing Jiancheng Bioengineering Institute, China.

### Determination of Hormone-Sensitive Lipase Activity

After blood collection, hens were euthanized to collect abdominal fat samples; the samples were placed in sample bags and rapidly frozen in liquid nitrogen for further analysis. The Bicinchoninic acid technique was used to quantify the total protein content in the tissue homogenate supernatant. 10 % tissue homogenate was prepared with PBS solution and mechanically homogenized while kept in an ice bath. The resulting mixture was centrifuged at 3000 rpm for 10 min at 4 °C to get the supernatant to measure the activity of hormone-sensitive lipase (**HSL**). The steps followed the instructions in the ELISA kit manual (Shanghai Enzyme Link Biotechnology Co, China).

### Liver Homogenate Biochemical Analysis

Liver samples were also collected from the slaughtered hens to prepare liver homogenate (10 %). The tissue samples used for the liver homogenate had a large amount of fat, so the homogenizing medium had to be switched to anhydrous ethanol for extraction. The liver homogenate was used to measure the concentrations of TC, TG, HDL-C, and LDL-C in the liver using commercially available kits according to the manufacturer's instructions (Nanjing Jianjian Bioengineering Institute, China). The ratio of HDL-C to LDL-C (HDL-C/LDL-C) was also calculated.

### Liver Histopathology

Liver tissue samples (1 cm) were collected from the left lobe tip and promptly placed in a 4 % paraformaldehyde solution for internal fixation. The tissue was then dehydrated using a gradient of alcohol, embedded in paraffin wax, and sliced to a thickness of approximately 4 μm using a paraffin slicer. Subsequently, the liver sections were stained with Hematoxylin and eosin (**H&E**). Similarly, liver blocks were OCT-embedded and subsequently cut into 8 μm-thick sections using a frozen sectioning machine. The sections were stained with Oil Red O satin. Both types of slides were examined under a light microscope and captured in photographs (Nikon Eclipse 80i, Nikon, Japan).

### Quantitative Real-Time Fluorescence PCR (qPCR)

A sample of liver tissue weighing 50 mg was collected, and its RNA was extracted using the Trizol technique (Monad Biotechnology Co., China). The concentration and purity of the RNA were determined using the NanoDrop 2000 Ultra-Micro Spectrophotometer (Thermo Fisher Scientific, America). Afterward, the entire RNA was transcribed in reverse per the guidelines of the reverse transcription kit (Monad Biotechnology Co, China), forming cDNA. The cDNA was promptly stored in a freezer at -20°C for future investigations. The primers were constructed using Primer Premier 5.0 software (Premier Canada, Inc. Detailed references are available on the website: https://www.premierbiosoft.com/primerdesign/). The primer details are presented in Table 3. The primers were synthesized by Beijing Tsingke Biotech Co, China.

**Table 3:** Primer sequences information.

Gene	Primer sequences (5’→3’)			Accession number
PPARγ	F: GCAGGAACAGAACAAAGAAG	20	373928	NM_001001460.2
	R: TGCCAGGTCACTGTCATCTA	21		
PPARα	F: TGTGGAGATCGTCCTGGTCT	20	374120	NM_001001464.1
	R: CGTCAGGATGGTTGGTTTGC	20		
LXRα	F: TGGAGAGACTACAGCACACCTATG	24	395221	NM_204542.3
	R: GTGAACACTACTTAGCGTCCGAAG	24		
SREBP1c	F: CCCGAGGGAGACCATCTACA	20	373915	NM_204126.3
	R: GGTACTCCAACGCATCCGAA	20		
CYP8B1	F: GGGTTACGCACTGGACTTCA	20	425055	NM_001005571.1
	R: GTAGCCAAAAACCCGGAGGA	20		
FXR	F: AGTAGAAGCCATGTTCCTCCGTT	23	373902	NM_001396910.1
	R: GCAGTGCATATTCCTCCTGTGTC	23		
β-actin	F: GCCAACAGAGAGAAGATGACACAG	24	396526	NM_205518.2
	R: CATCACCAGAGTCCATCACAATACC	25		

Real-time fluorescence qPCR was performed on liver tissues to measure the mRNA levels of Peroxisome proliferators-activated γ preceptor (**PPARγ**), Peroxisome proliferators-activated α preceptor (**PPARα**), Liver X receptor (**LXRα**), Sterol regulatory element binding protein-1c(**SREBP1c**), Sterol 12α-hydroxylase (**CYP8B1**), and Farnesoid X receptor (**FXR**) genes. The internal reference gene used was β-actin. The melting curve acquisition program was set up automatically by the qPCR instrument (30 s at 95 °C, then forty cycles of 10 s at 95 °C and 10 s at 60 °C, 30 s at 72 °C), and two replicate wells were made for each gene in each sample. The qPCR procedure was performed with reference to Wang et al. (2022). The Ct values obtained were averaged for data analysis, and the relative expression levels of the target genes were determined using the 2^-ΔΔCt^ method.

### Gut Morphology

The intestines were collected from the duodenum (near the end 1/3 of the stomach), jejunum (middle intestinal segment), and ileum (near the cecum 1/3). The method used for staining the duodenum, jejunum, and ileum was identical to the liver H&E staining approach. The Image-Pro Plus 6.0 program assessed the villus height and crypt depth. Subsequently, the ratio of villus height to crypt depth (**V/C**) was calculated. The microscope used was Nikon Eclipse 80i, Nikon, Japan.

### Short-Chain Fatty Acids Analysis

Cecum contents were collected from the slaughtered hens and were stored at -80°C till further analysis. Cecum contents (300 mg) and ultrapure water (1.5 mL) were mixed by shaking and left on ice for 1 h. Subsequently, it was centrifuged (10000 × g for 15 min at 4 °C). Carefully aspirated the supernatant, and 20 μL of 85 % orthophosphoric acid per 1 mL of supernatant was added and shaken to mix. After standing on ice for 1 h, the samples were centrifuged at 10,000 g for 15 min at 4 °C. The supernatant was injected through a 0.22 μm microporous membrane into a 1.5 ml injection vial. In addition, standards were used to develop the standard curves. The concentration of short-chain fatty acids (**SCFAs**) was determined by gas chromatography (Agilent 7890B, USA) using headspace injection. The chromatographic column used in the study was a DB-624 model with dimensions of 30 m in length, 0.32 mm in diameter, and 1.8 μm in particle size. The temperature was initially set at 100 °C for 5 min, followed by a gradual increase to 200 °C at a rate of 20 °C per minute. The temperature was then maintained at 200 °C for an additional 5 min. The detector was an FID flame ionization detector at 250 °C, the injection temperature was 200 °C, the injection volume was 25 μl, and the split ratio was 5:1.

### Microbiome Sequencing and Analyses

The cecum contents were also collected in a freezing tube for microbiome sequencing. Samples were frozen in liquid nitrogen and stored in a freezer at -80 °C. Based on the results of above blood serum biochemical analysis, hepatic lipid metabolism, and gut morphology, the ND and BG-4 groups with significant performance were chosen for a comparative study. Six samples were randomly selected from each group, and the 16S rDNA sequencing process and analytical methods for cecum contents have been described in detail in a previous study (Wang et al., 2024). A simplified experimental procedure and analytical method are presented here (performed by Novozymes Bioinformatics Co. Ltd. and its analytical platform, Tianjin, China). Total DNA was extracted from all groups of cecal coeliac samples using the QIAamp Fecal Rapid DNA Detection Kit (TianGen, China, Catalog #: DP712) according to the manufacturer's instructions. Total DNA was extracted from all groups of cecal coeliac samples using a Nanodrop 2000 (Thermo Scientific, Wilmington, DE), and a 1 % DNA concentration and quality were measured using Nanodrop 2000 (Thermo Scientific, Wilmington, DE) and 1 % agarose gel electrophoresis. Qualified DNA samples were amplified using bacterial primers specific to the 16S rRNA gene variable region V3-V4. Mix the same volume of 1X loading buffer (containing SYB green) with PCR products and operate electrophoresis on 2 % agarose gel for detection. PCR products were mixed in equidensity ratios. Then, the mixture of PCR products was purified with a Universal DNA Purification Kit (TianGen, China, Catalog #: DP214). Sequencing libraries were generated using NEB Next® Ultra™ II FS DNA PCR- free Library Prep Kit (New England Biolabs, USA, Catalog #: E7430L) following the manufacturer's recommendations and indexes. Manufacturer's recommendations and indexes were added. The PE 250 was then up-sequenced on a NovaSeq 6000.

The following analyses were analyzed using the company's QIIME2 software (Version QIIME2-202006). Alpha diversity (Chao1, Shannon's index, Simpson's index; T-test, or Wilcoxon rank-sum test according to the normal distribution of the samples) and beta diversity ((un)weighted_unifrac), MetaStat (Wilcoxon rank sum test or Fisher's test depending on the sample), T-test, and LEfSe analysis (t non-parametric factor Kruskal-Wallis and rank test, Wilcoxon test, and LDA). All other microbiota indicators were tested by T-test or Wilcoxon test according to their normal distribution. The 16S rRNA sequence data are available in the Sequence Read Archive (**SRA**) database under accession number PRJNA1195665.

### Statistical Analysis

Data were checked for normality with the Shapiro-Wilk test in SPSS 24.0. Normally distributed data were analyzed based on 1-way ANOVA, and the differences between means were compared using Tukey's post hoc test. Non-normal data were analyzed with the Kruskal−Wallis test or Wilcoxon test. Curvilinear regression analyses were performed using polynomial regression to examine the relationship between the BG additive level and the observed indicators. Quadratic regression analyses were conducted for the indicators with significant difference resulted from BG supplementation to predict the optimal level of BG. GraphPad Prism 8.0 software was used to generate appropriate bar and line graphs. The statistical results were reported as the Mean ± SEM. *P* < 0.05 was used to determine statistical significance, whereas *P* < 0.01 indicated extreme significance. If the *P* value fell between 0.05 and 0.10, it suggested a tendency towards significance. In microbial sequencing, * indicates the significance.

## RESULTS

### Serum Biochemical Analysis

**Fig. 1:**
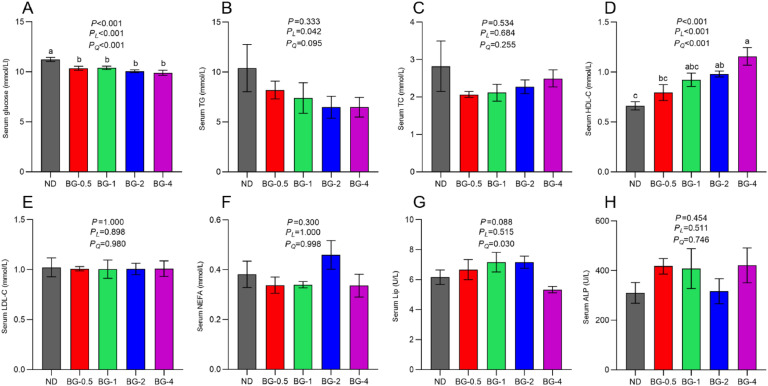
Effect of butyrate glycerides on serum glucose, TG, TC, lipid metabolism-protein, and lipase activities in laying hens. (A) serum glucose; (B) serum triacylglycerol; (C) serum total cholesterol; (D) serum HDL-C; (E) serum LDL-C; (F) serum NEFA; (G) serum Lipase activities; (H) serum ALP. Different superscripts (a, b and c) on the bar of different groups for the same treatments showed significant differences (*P* < 0.05). Polynomial regression was used to test the linear and quadratic relationship between butyrate glycerides supplemental levels. ND was a control group fed a basal diet, whereas BG-0.5, BG-1, BG-2 and BG-4 groups fed a basal diet supplemented with 0.5, 1, 2 and 4 g/kg of BalanGut LS P, respectively.

The results demonstrated that feeding BG (0.5 to 4 g/kg) led to a considerable decrease in serum Glu levels compared to the ND group (*P* < 0.001), confirming the hypoglycemic impact of BG (Fig. 1 A). The supplemetation of BG led to a noteworthy increase (*P* < 0.01) in HDL-C levels in the serum of laying hens, with the most favorable outcome observed in the group receiving 4 g/kg BG (*P* < 0.001, Fig. 1 D). Furthermore, the inclusion of BG had the tendency to elevate serum lipase activity in laying hens (*P* = 0.088, Fig. 1 G) and exhibited a significant quadratic correlation (*P* < 0.05) with the increasing in BG dosage level. However, it did not significantly affect (*P* > 0.05) TC, TG, ALP, LDL-C, and NEFA levels.

### Hormone-Sensitive Lipase Activity in Abdominal Fat

**Fig. 2:**
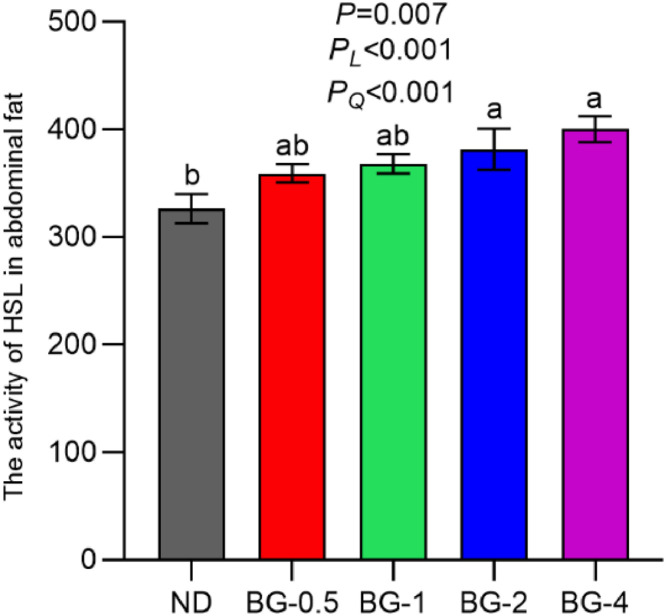
Effect of butyrate glycerides on HSL activity in abdominal fat of laying hens. Different superscripts (a, b and c) on the bar of different groups for the same treatments showed significant differences (*P* < 0.05). Polynomial regression was used to test the linear and quadratic relationship between butyrate glycerides supplemental levels. ND was a control group fed a basal diet, whereas BG-0.5, BG-1, BG-2 and BG-4 groups fed a basal diet supplemented with 0.5, 1, 2 and 4 g/kg of BalanGut LS P, respectively.

In comparison to the ND group, the inclusion of 2 g/kg (*P* < 0.05) and 4 g/kg (*P* < 0.01) of BG in the diet resulted in a significant increase in HSL activity in the abdominal lipids of laying hens. This increase demonstrated a linear and quadratic relationship with the increasing dose of BG (*P* < 0.001, Fig. 2).

### Lipid Deposition and Metabolism in the Liver

**Fig. 3:**
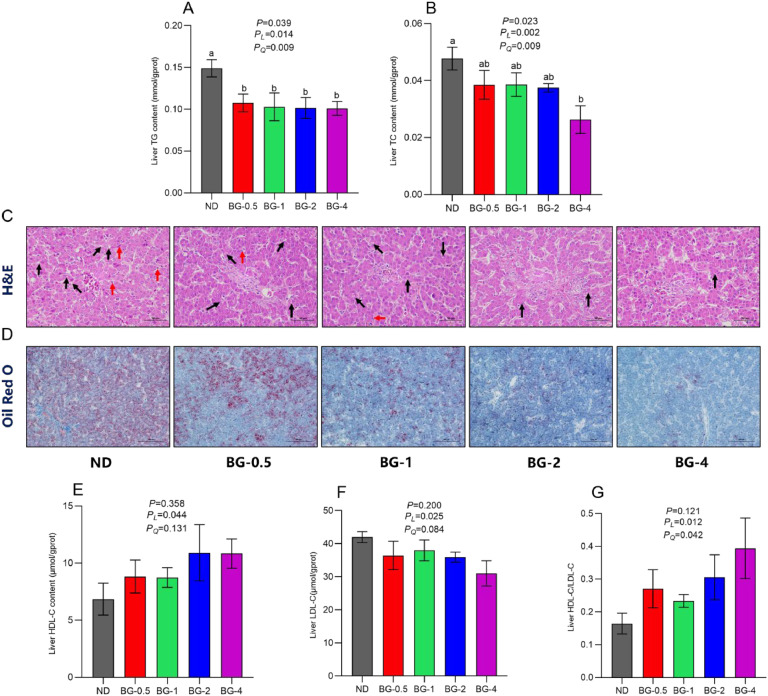
Effect of butyrate glycerides on lipid deposition and lipid metabolism-pritein in the liver of laying hens. (A) liver triacylglycerol; (B) liver total cholesterol; (E) liver HDL-C; (F) liver LDL-C; (G) liver HDL-C/LDL-C ratio. Different superscripts (a, b and c) on the bar of different groups for the same treatments showed significant differences (*P* < 0.05). Effect of butyrate glycerides on hepatic pathological canges in laying hens. (C) H&E staining of the liver (scale bar: 50 μm); (D) Oil Red O staining of the liver (scale bar: 100 μm). The black arrows in (C) point to hepatocyte lipid vacuoles, and red arrows point to focal infiltration of inflammatory cells. Polynomial regression was used to test the linear and quadratic relationship between butyrate glycerides supplemental levels. ND was a control group fed a basal diet, whereas BG-0.5, BG-1, BG-2 and BG-4 groups fed a basal diet supplemented with 0.5, 1, 2 and 4 g/kg of BalanGut LS P, respectively.

The TC and TG content of the liver experienced a substantial decrease in all groups treated with BG compared to the control group (*P* < 0.05, Fig. 3 A-B). Additionally, linear and quadratic changes were exhibited as the supplemental level of BG increased (*P* < 0.05). The BG-4 group exhibited that TG and TC contents decreased by 32.29 % and 44.84 %, respectively, than the ND group (*P* < 0.01). In addition, liver fat deposition was also analyzed through H&E staining and oil red O staining (Fig. 3 C-D). The results revealed that the liver of the ND group had a higher buildup of fatty vacuoles and lipid droplets than BG-supplemented groups. Additionally, there was evidence of inflammatory cell infiltration and erythrocyte accumulation.

No significant effect of BG supplementation was observed on hepatic HDL-C and HDL-C levels. However, hepatic HDL-C and HDL-C levels numerically increased and decreased in all BG-supplemented groups compared to the ND group (Fig. 3 E-F). In addition, there was a significant (*P* < 0.05) linear effect on increasing hepatic HDL-C and HDL-C/LDL-C ratio and decreasing hepatic LDL-C with increased dietary BG levels. Similarly, a significant (*P* < 0.05) quadratic effect on increasing hepatic HDL-C/LDL-C ratio was also observed with increasing dietary BG levels (Fig. 3 G).

### Expression of Genes Related to Hepatic Lipid Metabolism

**Fig. 4:**
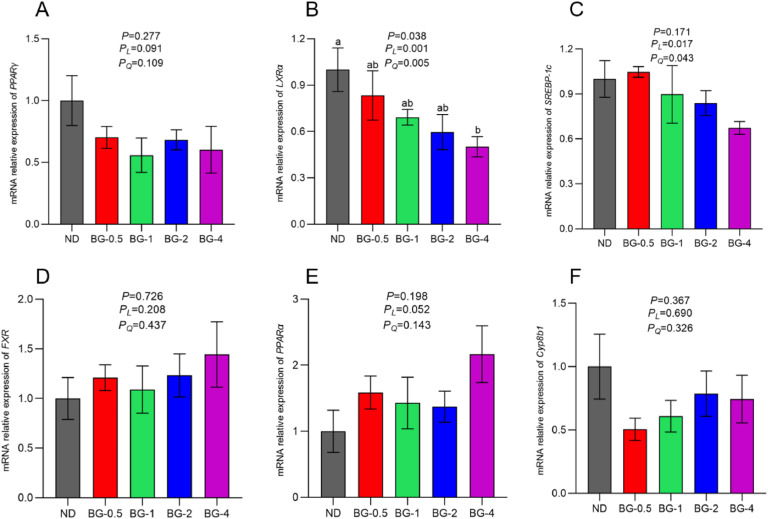
Effect of butyrate glycerides on the expression of genes related to lipid metabolism in the liver of laying hens. Relative mRNA expression of (A) PPARγ, (B) LXRα, (C) SREBP1c, (D) FXR, (E) PPARα, (F) Cyp8b1. Different superscripts (a, b and c) on the bar of different groups for the same treatments showed significant differences (*P* < 0.05). Polynomial regression was used to test the linear and quadratic relationship between butyrate glycerides supplemental levels. ND was a control group fed a basal diet, whereas BG-0.5, BG-1, BG-2 and BG-4 groups fed a basal diet supplemented with 0.5, 1, 2 and 4 g/kg of BalanGut LS P, respectively.

In this study, the mRNA expression levels of genes associated with hepatic lipid metabolism were analyzed to investigate the molecular mechanisms via which BG affects hepatic lipid metabolism during the late egg-laying stage. Fig. 4 B demonstrates that BG significantly reduced the mRNA expression of the liver X receptor α (LXRα), a gene associated with lipid production (*P* < 0.05). Moreover, the inhibitory effect of BG showed both linear and quadratic trends, which were statistically significant (*P* < 0.01) as the amount of supplemented BG increased. LXRα expression in the BG-4 group was significantly lower than in the ND group (*P* < 0.05). In addition, there was no significant difference in mRNA expression of PPARγ and SREBP-1c between the groups (*P* > 0.05, Fig. 4 A, 4 C). Nevertheless, BG tended to diminish PPARγ expression (*P* = 0.091) progressively and both linearly and quadratically suppress SREBP-1c mRNA expression (*P* < 0.05, Fig. 4 C). Fig. 4 F showed that the mRNA expression of Cyp8b1 was reduced in all BG groups compared to the ND group, whereas the mRNA expression of FXR and PPARα was raised. However, these changes were not statistically significant (*P* > 0.05, Fig. 4 D and E).

### Gut Morphology

**Fig. 5:**
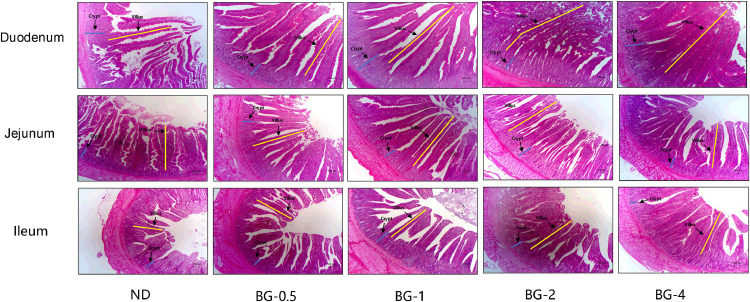
Small intestine morphology in laying hens supplemented with butyrate glycerides based on hematoxylin and eosin staining observed under 40x magnification. ND was a control group fed a basal diet, whereas BG-0.5, BG-1, BG-2 and BG-4 groups fed a basal diet supplemented with 0.5, 1, 2 and 4 g/kg of BalanGut LS P, respectively. Reference scale bar = 100 μm. Villus height = yellow line, crypt depth = blue line.

**Fig. 6:**
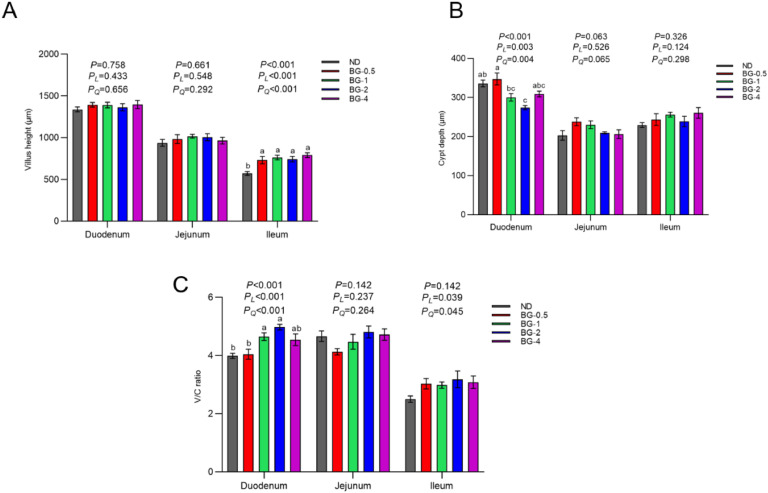
Effect of butyrate glycerides on morphology of intestinal mucosal of laying hens. (A) villus height; (B) crypt depth; (C) villus height/crypt depth ratio (V/C). Different superscripts (a, b and c) on the bar of different groups for the same treatments showed significant differences (*P* < 0.05). Polynomial regression was used to test the linear and quadratic relationship between butyrate glycerides supplemental levels. ND was a control group fed a basal diet, whereas BG-0.5, BG-1, BG-2 and BG-4 groups fed a basal diet supplemented with 0.5, 1, 2 and 4 g/kg of BalanGut LS P, respectively.

Fig. 5 demonstrates that the intestinal epithelial structure in the ND group was significantly more disturbed, characterized by crumpled and fractured intestinal villi and incomplete crypts at the bottom. Dietary supplementation of BG resulted in enhanced healing of intestinal villus damage. The small intestinal glands exhibited a well-structured arrangement, and the occurrence of villus-breaking symptoms was significantly decreased and aligned. According to Fig. 6 A–C, the duodenal villus height did not change significantly in all BG groups compared to the ND group (*P* > 0.05). However, there was a significant decrease in crypt depth and a significant increase in the ratio of villus height to crypt depth (V/C) (*P* < 0.01, Fig. 6 B-C). There was a trend of decreasing crypt depth in the jejunum (*P* = 0.063), but BG did not have a significant impact on villus height and V/C in the jejunum (*P* > 0.05). In addition, the addition of BG highly significantly increased the villus height of the ileum (*P* < 0.001) but had no significant influence on crypt depth and V/C (*P* > 0.05).

### The Concentration of SCFA in the Cecum Contents

**Fig. 7:**
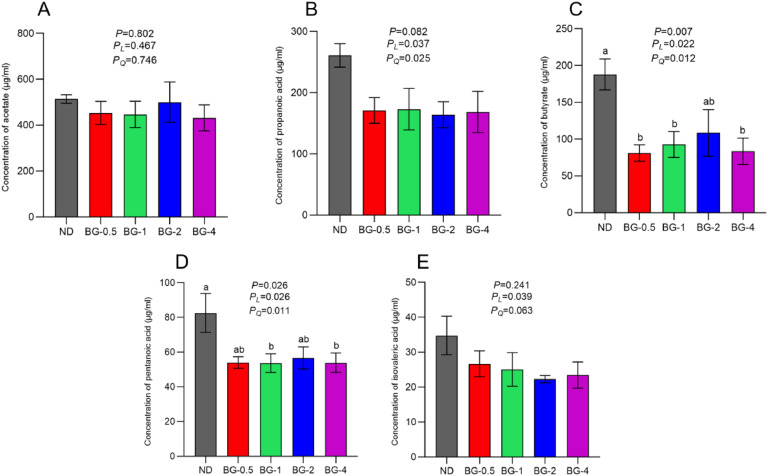
Effect of butyrate glycerides on SCFAs in cecal content of laying hens. (A) concentration of acetate; (B) concentration of propanoic; (C) concentration of butyrate; (D) concentration of pentanoic acid; (E) concentration of isovaleric acid. Different superscripts (a, b and c) on the bar of different groups for the same treatments showed significant differences (*P* < 0.05). Polynomial regression was used to test the linear and quadratic relationship between butyrate glycerides supplemental levels. ND was a control group fed a basal diet, whereas BG-0.5, BG-1, BG-2 and BG-4 groups fed a basal diet supplemented with 0.5, 1, 2 and 4 g/kg of BalanGut LS P, respectively.

The dietary supplementation of BG in laying hens tended to decrease the concentration of propionic acid in their cecum contents (*P* = 0.082; Fig. 7 B). Furthermore, there was a linear and quadratic decrease in propionic acid concentration with increasing BG dose (*P* < 0.05). The levels of n-butyric acid (in BG-0.5, BG-1, and BG-4 group) and pentanoic acid (BG-1 and BG-4 groups) in the cecum contents of the BG groups were considerably reduced (*P* < 0.05; Fig. 7 C-D) than the ND group. Furthermore, there was a strong linear and quadratic correlation (*P* < 0.05) between the amount of BG supplemented and the decrease in the concentrations of these acids. However, adding BG to the diet did not affect (*P* > 0.05) the acetic acid and isovaleric acid concentrations (Fig. 7 A, 7 E).

### Prediction for Optimal Level of BG Supplementation

Quadratic regression analyses were conducted for the indicators in serum, liver, and intestine with significant difference resulted from BG supplementation to predict the optimal level. Table 4 showed the quadratic regression equations for the indicators, and the optimal amount of BG supplementation to the diet ranges from 2.45 to 4 g/kg.

**Table 4:** Analysis of quadratic regression equations.

	Indicator	Quadratic regression equation	Optimal BG level (g/kg)
Serum	Glucose	Y = 11.04-0.81X+0.13X^2^	3.12
	HDL-C	Y = 0.68+0.22X-0.03X^2^	3.67
	Lipase activity	Y = 6.17+1.26X-0.37X^2^	4.00
HSL	HSL activity	Y = 3.33E^2^+37.63X-5.27X^2^	3.57
Liver	Triacylglycerol	Y = 0.14-0.04X+7.18E^-3^X^2^	2.79
	Total cholesterol	Y = 0.04-5.34E^-3^X+2.07E^-4^X^2^	4.00
	HDL-C/LDL-C ratio	Y=0.19+0.07X-5.22E^-3^X^2^	4.00
mRNA expression	SREBP1c	Y = 1.03-0.1X+2.6E^-3^X^2^	4.00
	LXRα	Y=0.98-0.3X+0.04X^2^	3.75
Morphology of intestinal	Ileum villus height	Y = 6.19E^2^+1.3E^2^X-22.47X^2^	2.89
	Duodenum crypt depth	Y = 3.5E^2^-57.81X+11.78X^2^	2.45
	Ileum V/C	Y = 2.61+0.51X-0.1X^2^	2.55
	Duodenum V/C	Y = 3.87+0.89X-0.18X^2^	2.47
Optimal BG level range:2.45-4.00 g/kg			

### Cecal Microbiome Diversity, Composition, and Sequencing

**Fig. 8:**
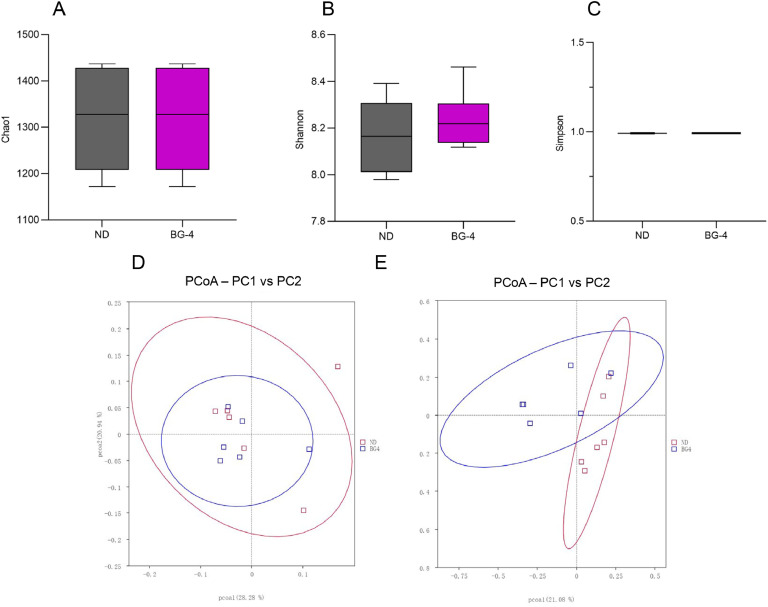
Effect of butyrate glycerides on the microbial diversity in cecal content of laying hens. (A) Chao1 index; (B) Shannon index; (C) Simpson index; (D) PCoA analysis based on weighted unifrac distance; (E) PCoA analysis based on unweighted unifrac distance. * represents significant differences among each group (*P* < 0.05). ND was a control group fed a basal diet, whereas BG-4 group fed a basal diet supplemented with 4 g/kg of BalanGut LS P.

To further examine the impact of glycerol monobutyrate on the intestinal microbiota of laying hens during the late-laying period, 16S rRNA sequencing on samples of cecum content taken from the hens was analyzed. Based on the outcomes of the previous tests, the control group and the group with the highest dose of BG-4, which showed a more efficient intervention in fatty liver, were chosen for comparison study using 16S rRNA analysis. In Fig. 8, both groups showed similar results in terms of Alpha diversity (Chao1, Shannon, Simpson indices) and Beta diversity (PcoA) between the two groups.

**Fig. 9:**
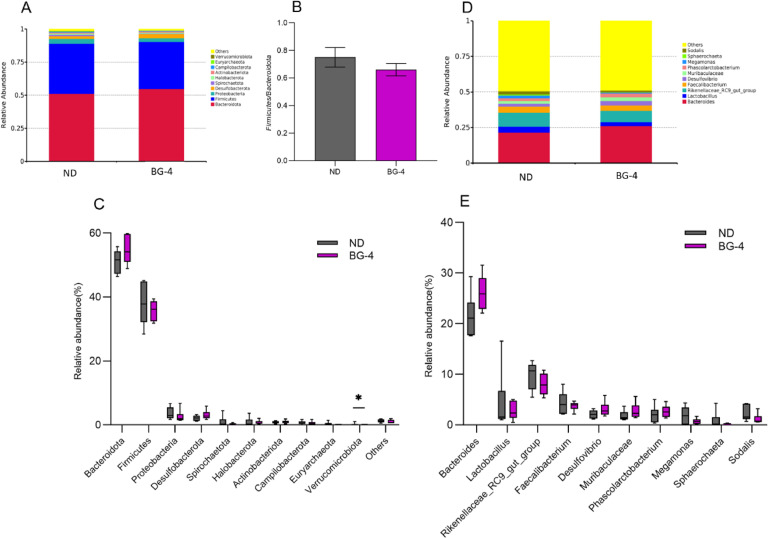
Effect of butyrate glycerides on the composition of cecal microbiota of laying hens. (A) Microbial community barplot at phylum level; (B) The ratio of relative abundance of Firmicutes to Bacteroidota; (C) Microbial differences at the phylum level; (D) Microbial community barplot at genus level; (E) Microbial differences at genus level. * represents significant differences among each group (*P* < 0.05). ND was a control group fed a basal diet, whereas BG-4 group fed a basal diet supplemented with 4 g/kg of BalanGut LS P.

The top 10 most abundant bacterial communities in the cecum flora of each group of samples were analyzed based on phylum and genus levels, respectively. The results shows that the presence of Verrucomicrobiota was significantly higher in the ND group than in the BG-4 group. Bacteroidetes, Firmicutes, and Proteobacteria dominated both groups at the phylum level (Fig. 9 C). Fig. 9 C also demonstrates that the BG group had a slightly higher abundance of Bacteroidetes and a slightly lower abundance of Proteobacteria compared to the ND group. The Firmicutes/Bacteroidetes ratio decreased with adding BG but did not reach a significant level. At the genus level, the BG treatment tended to increase the presence of *Bacteroides* (*P* = 0.074) compared to the ND group. In addition, the BG-4 group showed a decreased abundance of *Lactobacillus, Rikenellaceae_RC9*_gut_group, *Faecalibacterium, Megamonas, Sphaerochaeta*, and *Sodalis*. On the other hand, there was an increase in the abundance of *Desulfovibrio, Muribaculaceae*, and *Phascolarctobacterium*. However, these changes were not reached a significant level (Fig. 9 E).

**Fig. 10:**
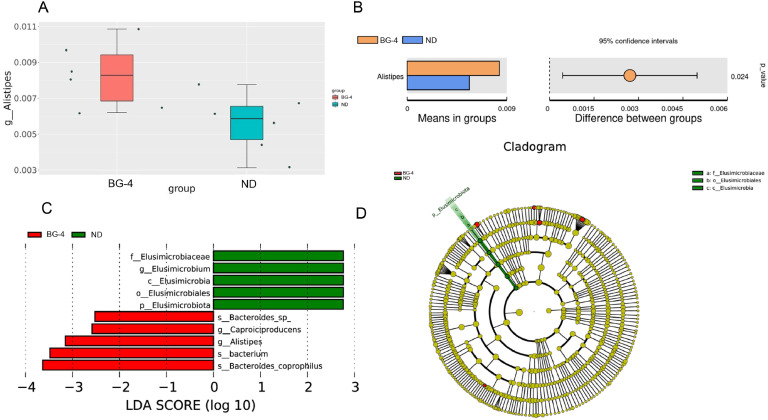
Effect of butyrate glycerides on T-test-Qiime2 and LEfSe analysis of microbiota in cecum content of laying hens. (A) *Alistipes* MetaStat analysis; (B) *Alistipes* T-test analysis; (C) LEfSe analysis distribution histogram with LDA score of greater than 2.5; (D) Cladogram generated from LEfSe analysis. MetaStat can only plot box plots of the abundance distribution of species with differences between groups. The right-hand side of Figure B shows the p-value of the T-test, *P* < 0.05. ND was a control group fed a basal diet, whereas BG-4 group fed a basal diet supplemented with 4 g/kg of BalanGut LS P.

T-test and LefSe analyses were performed at the genus level (Fig. 10). It was found that the abundance of *Alistipes* was significantly increased in the BG-4 group compared to the ND group (*P* < 0.05, Fig. 10 B). The ND group was significantly enriched with *Elusimicrobiota*. In contrast, the BG-4 group had higher levels of *Bacteroides_coprophil*us, *bacterium, Alistipes, Caproiciproducens*, and *Bacteroides_sp*.

## DISCUSSION

Literature suggested that glycerol monobutyrate can enhance broilers' muscle and abdominal lipid accumulation (Bedford et al., 2017; Bedford et al., 2018). Additionally, it has been observed that glycerol monobutyrate can decrease liver fat and succinic acid levels in obese rats by mitigating lipid metabolism abnormalities (Nguyen et al., 2017; Nguyen et al., 2019; Nguyen et al., 2020). Therefore, in this study, glyceryl monobutyrate was supplemented to the diet of laying hens during the late laying phase. The purpose was to examine how BG affects lipid metabolic homeostasis and to identify the optimal dosage level.

The liver plays a crucial role in lipid metabolism, with over 90 % of fatty acid synthesis in chickens occurring in this organ. Due to lipid biosynthesis and catabolism imbalance, excessive fat accumulation in the liver leads to hepatomegaly (Kirpich et al., 2015). The levels of TC, TG, HDL-C, and LDL-C in the liver are commonly utilized to assess disturbances in lipid metabolism (Klop et al., 2013). The present study's findings indicate that dietary supplementation of BG to laying hens for 5 weeks resulted in a considerable decrease in hepatic TC and TG levels. This suggests that glycerol monobutyrate can potentially alleviate hepatic fat deposition effectively. Accumulation of hepatic fat due to abnormally elevated blood lipids causes fatty lesions in the parenchymal cells of the liver. The liver exhibits pathological alterations, including surface swelling and congestion. Histological analysis of the fatty liver reveals a significant presence of lipid droplets and fat vacuoles inside the tissue sections, accompanied by the infiltration of inflammatory cells and the aggregation of red blood cells. Similar results were observed by Liu et al. (2023a) in laying hens with early hepatic steatosis. The findings from the H&E and Oil Red O staining in this study provide additional evidence that the development of fatty liver in laying hens during the late laying period results in structural harm to the liver. However, dietary supplementation with BG reduced hepatic lipid accumulation and improved liver health, particularly at the supplemental levels of 2 and 4 g BG per kg diet. This suggests that BG has the potential to treat FLHS in laying hens.

HSL is a key rate-limiting enzyme in lipolysis (Sato et al., 2010). Mutations in HSL in humans lead to a decrease in the ability to store lipids (Ong et al., 2011; Althaher, 2022). Anthonsen et al. (1997) have documented the presence of HSL activity in the adipose tissue of chickens. The current study found that the activity of HSL in the abdominal fat of laying hens was significantly higher in the BG groups (BG-2 and BG-4). Among them, the group that received the highest dose (4 g/kg) had the most favorable outcomes. Serum lipid and lipoprotein concentrations reflect the regulation of fatty acid cycling between the liver and peripheral tissues. LDL-C functions as the primary transporter of fatty acids (**FAs**) and cholesterol from the liver to tissues outside the liver. In contrast, HDL-C transports FAs and cholesterol from peripheral tissues back to the liver for breakdown (Jacobson et al., 2012). Our results showed that glycerol monobutyrate improved serum glycolipid levels during the late egg-laying period. This was achieved by decreasing serum glucose concentration and boosting HDL-C content and lipase activity.

The equilibrium between lipogenesis and lipolysis primarily influences lipid accumulation in animals. To gain a deeper understanding of how glycerol monobutyrate influences the regulation of lipid metabolism in the liver, the mRNA levels of genes involved in this process were analyzed. During lipogenesis, ACC is the first rate-limiting enzyme in the synthesis of fatty acids and is a crucial determinant of the maximum capacity for fat production (Lu et al., 2019). SREBP-1c functions as a nuclear transcription factor that binds to sterol regulatory elements and directly promotes the expression of the target gene ACC. It also stimulates the production of FAs and their integration into triglycerides (Horton et al., 2002; Wang and Tontonoz, 2018). LXRα is a ligand-activated nuclear receptor with essential functions in the transcriptional control of FAs synthesis by promoting SREBP-1c expression and activating the ACC and FAS promoters (Wang and Tontonoz, 2018). PPARγ is involved in the adipogenic process by enhancing the transcription of its target gene, SREBP-1c (Mirza and Sharma, 2019). Yang et al. (2023) treated ginkgo biloba extract to laying hens with FLHS, and the expression level of LXRα was significantly down-regulated in the treatment group. One study even indicated that overexpression of the miR-216/miR-217 cluster or reduction in TM9SF3 levels led to activation of the proliferator-activated receptor/sterol regulatory-element binding protein (PPAR/SREBP) pathway, thereby alleviating fatty liver in laying hens(Zhu et al., 2022). The findings of our study indicate that the levels of hepatic PPARγ, LXRα, and SREBP-1c were decreased in all groups supplemented with BG. This suggests that BG may decrease lipid accumulation in the liver during the late stage of laying hens by suppressing genes related to lipid synthesis in the liver through the PPARγ - LXRα - SREBP1c pathway.

Highly expressed FXR in the intestinal-hepatic tissues allows it to interact with the signaling molecule bile acid (**BA**), which influences BA homeostasis and the equilibrium of human lipid metabolism. When FXR is activated, it can decrease hepatic lipid levels by positively regulating the expression of PPARα (Rajani and Jia, 2018). PPARα is responsible for the liver's absorption and β-oxidation of FAs (Tateno et al., 2015). However, when the normal expression of FXR is disrupted, it, in turn, induces lipid accumulation in the serum and liver (Sinal et al., 2000). No significant impact on the expression of lipid transport and oxidation-related genes PPARα and FXR was observed in the BG group. This suggests that BG may not have the ability to regulate lipid metabolism by enhancing lipid oxidation.

The small intestine is the primary site of digestion and absorption of nutrients. A more mature and well-developed intestinal mucosal cellularity, intestinal digestion and absorption, and a more remarkable ability to resist invasion by hazardous pathogens can be identified by longer villi, shallower crypts, and a higher ratio of villus height to crypt depth (Xu et al., 2022). The present study demonstrated notable mucosal damage in the duodenum, jejunum, and ileum of laying hens during the later stages of production after intensive metabolism. However, the supplementation of BG in the diet effectively mitigated villi damage, reduced the crypt depth, and increased both villi height and the ratio of the villus height to the crypt depth. Li et al. (2015) found that supplemental glycerol tributyrate reduced the release of proinflammatory cytokines and improved intestinal morphology in broilers' duodenum, jejunum, and ileum, which is consistent with our results.

The intestines and the liver are anatomically and physiologically closely linked, known as the intestinal-liver axis. The microbiota plays a significant role in the gut-liver axis, impacting the gut, liver, and other organs through somatic circulation. Therefore, maintaining a symbiotic equilibrium between the microbiota and the gut is essential for optimal production performance (Celi et al., 2019; Bindari and Gerber, 2022). Since gut bacteria play an essential role in forming non-alcoholic fatty liver disease (**NAFLD**) in humans and rodents, we hypothesized that gut bacteria are also strongly associated with the development of FLHS in laying hens. In this study, it has been shown that hepatic fat deposition occurs in late-laying hens under conventional rearing conditions. Additionally, it has been observed that BG-4 reduced the hepatic fat deposition and intestinal morphology and structure. Consequently, the cecum contents from the ND and BG-4 groups were selected for 16S rRNA sequencing in the highly variable region of V3-V4.

Nutrients are carried through the upper intestine for only 2.5 h but remain in the cecum, which has the most significant bacterial biodiversity, for 12-20 h (Sergeant et al., 2014; Yan et al., 2017). Therefore, cecum contents are more representative in studying microbial-fatty liver associations. The study's findings indicate no statistically significant variation in the diversity of cecum intestinal flora between the ND and BG-4 groups that exhibited fatty liver. Similarly, Li et al. (2020) found that hepatic steatosis in laying hens is not influenced by the variety of intestinal flora but rather by the quantity. Specifically, a decrease in beneficial bacteria and an increase in harmful bacteria in the intestinal tract of laying hens can contribute to the development of hepatic steatosis, leading to a higher prevalence of FLHS. So, microbial structures were further analyzed at the phylum level.

The most dominant phylum in the cecum of laying hens are Bacteroidetes, Firmicutes, and Proteobacteria (Li et al., 2020). Bacteroidetes produces acetic, isovaleric, and succinic acids as the primary by-products of anaerobic respiration. These acids can serve as nutrition for other gut bacteria. Yoshida et al. (2021) reported that Bacteroides can be used to treat obesity and relieve defective catabolism of branched-chain amino acids in brown adipose tissue in mice with dietary obesity. Increased abundance of Firmicutes disrupts host energy metabolism and promotes fatty liver (Armougom et al., 2009). Several studies have indicated that an increased ratio of Firmicutes to Bacteroidetes abundance is typical of obesity and strongly associated with NAFLD severity (Indiani et al., 2018). In addition, alterations in Proteobacteria abundance are both a key determinant of gut health in animals and one of the hallmarks of intestinal flora dysbiosis (Shin et al., 2015). Proteobacteria enrichment may cause mice to develop obesity and other diseases resulting from low-grade inflammation (Zou et al., 2023). In our results, although there was a trend toward an increased abundance of Bacteroides at the genus level only in the BG-4 group, the correlative changes of decreased abundance of Firmicutes and Proteobacteria, increased abundance of Bacteroidetes (and *Bacteroides*), as well as a decrease in the ratio of Firmicutes/ Bacteroidetes (**F/B**) may at least partially explain the decrease in hepatic fat deposition in laying hens due to the addition of glycerol monobutyrate. Jia et al. (2023) treated cinaciguat to mice with lipid metabolism disorders, significantly decreased the F/B ratio, and increased the abundance of *Alistipes*, suggesting that *Alistipes* may mitigate lipid deposition by lowering triglycerides. This is in accordance with our results.

*Alistipes* is a relatively new sub-branch genus of Firmicutes. Comparative evidence shows that *Alistipes* may benefit liver fibrosis, cirrhosis, colitis, cancer immunotherapy, and cardiovascular disease (Sung et al., 2019; Parker et al., 2020). Additionally, its metabolic processes result in the production of propionic and acetic acids (Polansky et al., 2016). One study also demonstrated a decrease in F/B and an increase in the relative abundance of *Muribaculaceae* and *Alistipes* after treatment of lipid metabolism disorders in High-fat diet (**HFD**) mice (Fu et al., 2022). Liu et al. (2023b) also found that *Alistipes* were associated with alleviating fat deposition and inhibiting the value-addition and differentiation of fat molecules in broilers. The study's findings indicate that *Alistipes* was notably increased in the BG-4 group (*P* < 0.05). This suggests that BG can mitigate fatty liver in laying hens by enhancing the relative abundance of the beneficial bacterium *Alistipes* in the cecum. There are fewer studies on the relevance of *Elusimicrobiota* to lipid metabolism, and the limited literature suggests that patients with cirrhosis have a lower abundance of *Elusimicrobiota* compared to a healthy group (Sarangi et al., 2017). *Elusimicrobium* may be a potential dominant bacterium and biomarker for reducing blood glucose and insulin resistance in rats (Wang et al., 2021; Zhang et al., 2021). On the other hand, the opposite conclusion has also been made: the abundance of Elusimicrobia was higher in the type 2 diabetic rat model group than in the control group, and the abundance decreased after treatment (Gu et al., 2017). Verrucomicrobia was positively correlated with obesity indicators by Spearman's analysis, suggesting its detrimental role in obesity prevention by inhibiting hepatic fatty acid catabolism(Guo et al., 2021). Our sequencing results aligned with Turnbaugh et al. (2009), Li et al. (2016), and Gao et al. (2023), who reported higher levels of Verrucomicrobia in disordered lipid metabolism subjects. Therefore, we believe that supplemental BG reduces the abundance of harmful bacteria in late-laying hens and helps to prevent and mitigate fat deposition in laying hens.

According to certain studies, there is a notable rise in the presence of *Bacteroides_coprophilus* in obese people and patients with polycystic ovarian syndrome (Torres et al., 2018; Sun, 2019). However, there are also literature reports claiming that *Bacteroides_coprophilus* is a helpful bacterium in the intestines. The analysis of the Macrogenomic strain (**MGS**) revealed that *Bacteroides_coprophilus* was found in higher abundance in the intestinal tract of low-fat broilers compared to high-fat lineage broilers (Huo, 2015). Furthermore, the relative abundance of *Bacteroides_coprophilus* was notably reduced in patients with cirrhosis and gestational diabetes mellitus (Ren, 2020; Huang, 2022). We believe these differences may be attributed to differences in diet composition, feeding conditions, and pathological and physiological states. Zhang et al. (2019) proposed that Chinese herbs could hinder the progression of cardiovascular disease in pigs with high-fat diet-induced metabolic disorders. This was achieved by increasing the abundance of *Caproiciproducens* and improving the gut microbiota. Additionally, microbiological analyses showed that *Caproiciproducens* is the primary producer of hexanoic acid (Tang et al., 2022). In this study, LEfSe results showed that the ND group was significantly enriched for Elusimicrobiota, and the BG-4 group was enriched for *Bacteroides_coprophilus, bacteriuim, Alistipes, Caproiciproducens,* and *Bacteroides_sp.* Except for controversial genera, the addition of BG to the ration did reshape the composition of the intestinal bacteria in laying hens during the later phases of egg production, with significant enrichment of the beneficial bacteria such as *Bacteroidetes, Alistipes,* and *Caproiciproducens* in the cecum.

The importance of SCFA in regulating lipid metabolism disorders has gained increasing recognition in recent years. Acetate and butyrate are significant substances for lipid metabolism; propionate, produced from the intestines, greatly stimulates gluconeogenesis (den Besten et al., 2013). Interestingly, we observed that the level of SCFAs was notably lower in the group that received BG compared to the control group. We believe this may have happened for two reasons. First, higher concentrations of SCFAs in the ND group may originate from lipolysis in the ration. The more severe damage to the intestinal mucosa led to the buildup of SCFAs in the cecum, which was not rapidly absorbed and utilized. Second, although the variability of bacteria at the phylum and genus level has not yet reached significance, the ND group exhibited a higher abundance of the phylum *Thickettsia, Rikenellaceae_RC9_*gut_group, the genus *E. faecalis,* and *Giantomonas*, resulting in a greater combined production of SCFAs (Barcenilla et al., 2000; Duncan, 2002; Pryde, 2002; Kim et al., 2014; Fu et al., 2022). Furthermore, although there were significantly more *Alistipes* in the BG group than in the ND group at the genus level, the highest abundance of Avg remained low, and the levels of acetate and propionate produced were still low. Yin's team reported that *Alistipes* and Bacteroides play a significant role in acetic acid production in mouse feces (Yin et al., 2018). This may further explain why the content of SCFAs, such as acetic acid, in the cecum contents of the BG group, was lower than that of the ND group in this experiment. Despite today's prevailing view that increased levels of SCFAs have beneficial effects on animal lipid metabolism, several studies have proposed the "energy-harvesting" hypothesis (Morrison and Preston, 2016). This hypothesis suggests that SCFAs provide additional calories through fermentation in obese individuals, leading to explanations for weight gain. Consistent with this, research in mice has shown that the microbiota of obese individuals produce excessive SCFAs, which increases energy availability in the colon and leads to obesity (Bedford et al., 2017; Bedford et al., 2018). However, it also has been shown that the gut flora of obese individuals produce twice as many SCFAs as non-obese individuals, with propionate accounting for the most significant proportion (Schwiertz et al., 2010; Crovesy et al., 2020). Under physiological conditions, SCFAs such as acetic acid and propionic acid inhibit lipolysis in mice (Hong et al., 2005). This implies that increased SCFAs are at risk of enhancing fat deposition. To summarize, our findings indicate that including BG in the diet may have mitigated the metabolic risk factors associated with higher SCFAs levels in individual laying hens with fatty liver.

The main objective of current study was to investigate the impact of gradient levels of BG on lipid metabolism, intestinal mucosa morphology, and intestinal flora in laying hens during the late laying phase. From a commercial perspective, the optimal dosage and its improvement in performance and quality are the key points of concern for laying hens breeding enterprises. By performing quadratic regression analysis of serum, liver, and intestinal indicators that improved significantly after BG supplementation, it was found that the optimal amount of BG supplementation to the diet ranges from 2.45 to 4 g/kg. And in our experiment, laying performance was also improved significantly (data was included in an accepted paper of Chinese Journal of Animal Science, No.20240805-6). The egg production was linearly increased, and feed egg ratio was linearly decreased with the supplementation of BG. The laying performance of hens in 4 g/kg BG group is also the best, egg production was increased from 83.5 % to 87.9 % and feed egg ration decreased from 2.3 to 2.16. So that, under the conditions of this experiment, late-laying hens with 4 g/kg BG supplementation performed best overall.

## CONCLUSION

The supplementation of butyrate glycerides in the diet has been observed to enhance lipid metabolism in laying hens during the later stages of egg production. This was evidenced by a reduction in serum and hepatic lipid deposition, potentially modulating lipid metabolism via the PPARγ-LXRα-SREBP1c pathway. Additionally, dietary supplementation of BG has demonstrated the ability to enhance intestinal morphology and regulate the composition of intestinal flora in laying hens during the late stage of laying. Under the conditions of this experiment, the addition of 4 g/kg BG resulted in the best performance for late-laying hens. However, further research is necessary to elucidate the specific mechanisms underlying the elevation of cecal SCFAs concentrations.

## DISCLOSURES

We declare that we have no financial and personal relationships with other people or organizations that can inappropriately influence our work, there is no professional or other personal interest of any nature or kind in any product, service and/or company that could be construed as influencing the position presented in, or the review of, the manuscript entitled, ‘Effects of dietary supplementation with butyrate glycerides on lipid metabolism, intestinal morphology, and microflora population in laying hens’.
